# Prevalence of Extensively Drug Resistant Tuberculosis among Archived Multidrug Resistant Tuberculosis Isolates in Zimbabwe

**DOI:** 10.1155/2014/349141

**Published:** 2014-05-20

**Authors:** Tichaona Sagonda, Lucy Mupfumi, Rumbidzai Manzou, Beauty Makamure, Mqondisi Tshabalala, Lovemore Gwanzura, Peter Mason, Reggie Mutetwa

**Affiliations:** ^1^Biomedical Research and Training Institute, Harare, Zimbabwe; ^2^Department of Medical Laboratory Sciences, University of Zimbabwe, Harare, Zimbabwe; ^3^Immunology Department, College of Health Sciences, University of Zimbabwe, P.O. Box A178, Avondale, Harare, Zimbabwe

## Abstract

We conducted a cross-sectional study of second line drug resistance patterns and genetic diversity of MDR-TB isolates archived at the BRTI-TB Laboratory, Harare, between January 2007 and December 2011. DSTs were performed for second line antituberculosis drugs. XDR-TB strains were defined as MDR-TB strains with resistance to either kanamycin and ofloxacin or capreomycin and ofloxacin. Strain types were identified by spoligotyping. No resistance to any second line drugs was shown in 73% of the isolates, with 23% resistant to one or two drugs but not meeting the definition of XDR-TB. A total of 26 shared types were identified, and 18 (69%) matched preexisting shared types in the current published spoligotype databases. Of the 11 out of 18 clustered SITs, 4 predominant (>6 isolates per shared type) were identified. The most and least abundant types were SIT 1468 (LAM 11-ZWE) with 12 (18%) isolates and SIT 53 (T1) with 6 (9%) isolates, respectively. XDR-TB strains are rare in Zimbabwe, but the high proportion of “pre-XDR-TB” strains and treatment failure cases is of concern. The genetic diversity of the MDR-TB strains showed no significant association between SITs and drug resistance.

## 1. Introduction

Tuberculosis (TB) is second to human immunodeficiency virus (HIV) as the leading cause of death due to a single infectious agent in the world. Although the global prevalence of TB has been on the decline (from 13 million in 2010 to 12 million in 2011) [1], the burden of TB that remains is still very large. Geographically, America and Europe contribute only 7.3% of the total global burden, largely made possible by huge financial, infrastructural, and manpower resources channeled towards control efforts. However in resource limited settings the picture is drastically different. Asia and Africa contributed 59% and 29% of the global TB burden, respectively. In Asia, India and China have the highest TB burden with the two combined accounting for 38% [1] of the global TB burden in 2011. In the African region, 9 countries (South Africa, Zimbabwe, DR Congo, Tanzania, Ethiopia, Kenya, Nigeria, Uganda, and Mozambique) are on the 22 high TB burden list with South Africa having the highest TB burden in the region in 2011. During the same period, HIV coinfection was estimated to be 13% of the global TB prevalence, with Sub-Saharan Africa accounting for 80% [1] of this burden. Deaths related to TB/HIV coinfection were estimated to be 0.4 million, about a third of the total TB related deaths reported in 2011 [1].

Extensively drug resistant TB is defined as multidrug resistant TB (MDR-TB) plus resistance to a fluoroquinolone and at least one of three injectable second line drugs (amikacin, kanamycin, or capreomycin). XDR-TB infections are very difficult to treat and characterized by high mortality. As with any other public health problem, the emergence and spread of XDR-TB are thought to be due to many factors, though shortage of human resources, inappropriate health infrastructure, and insufficient medicines are probably the more significant causes. Globally the number of countries identifying XDR-TB increased from 58 in January 2010 [2] to 84 by the end of 2011 [1]. In 2008 a total of 23 760 XDR-TB cases were reported, with the number increasing to 27 900 in 2011.

Current TB global data estimates that 9.0% of all multidrug resistant TB (MDR-TB) cases are XDR-TB. Former States of the Soviet Union, India, and China have the greatest burden of XDR-TB globally [3–5], with Estonia reporting an XDR-TB burden of 18.7% among MDR-TB cases [1]. In Sub-Saharan Africa, 1st line drug sensitivity test (DST) coverage is very poor and 2nd line DST coverage is virtually nonexistent at national level. In 2011, it was estimated that only 0.2% of the 2.3 million new TB cases reported in Sub-Saharan Africa had a DST result [1]. To date only 16/53 African countries have reported at least one case of XDR-TB, the majority of which were from independent research activities rather than nationally acquired data. South Africa currently accounts for the highest XDR-TB in the region with outbreaks being reported in various provinces since 2006 [1, 6].

To date Zimbabwe has not reported any cases of XDR-TB, which is unusual given that four of her neighbors had reported at least one case of XDR-TB by late 2011 [1, 7]. Given the large population movements between Zimbabwe and her neighbors that resulted from the Economic Crisis of 2006–2008, it is expected that XDR-TB be reported in Zimbabwe. The lack of data on XDR-TB trends in the country could be a result of the poor DST coverage (currently 0.8 per 5 million) and the lack of second line DST in our TB culture laboratories [1]. Currently, there are two laboratories with TB culture facilities: The National TB Reference Laboratory (NTBRL) in Bulawayo and The National Microbiology Reference Laboratory (NMRL), TB section, in Harare.

## 2. Materials and Methods 

### 2.1. Study Design

This was a cross-sectional study where all available archived MDR-TB isolates were retrieved from the BRTI/NMRL TB Laboratory for further analysis.

### 2.2. Isolate Source

A record review of laboratory DST records was done to find MDR-TB isolates identified at the BRTI/NMRL TB Laboratory between January 2007 and December 2011. The list of MDR-TB isolates generated from the record review was used to locate and retrieve the archived isolates form the laboratory repository. Only the first MDR isolate of a patient was included, excluding multiple isolates form the same patient. All of the available isolates were retrieved. Another record review of “request for examination” forms submitted to the laboratory along with the original patient specimen was done to collect patient demographics. Data on age, gender, TB treatment history, and province of origin were collected using a coded form.

### 2.3. MDR-TB Isolate Recovery from Storage Media

Isolates were retrieved from the freezer and allowed to thaw to room temperature. Two loops full of the Trypticase Soy Broth (TSB), per isolate, were inoculated onto plain LJ slopes and incubated at 37°C. Slopes were incubated for a maximum of 8 weeks and checked weekly for growth. Pure colonies of positive isolates were harvested by scraping pure colonies of MTB from LJ slopes to use them as inoculum for 2nd line DST. The remaining pure colonies were harvested and stored in 0.5 mL of sterile distilled water at −80°C for later use in DNA extraction.

### 2.4. Second Line DST on 7H10 Middlebrook Agar

Second line DST of revived isolates was performed using the proportion method on 7H10 Middlebrook agar [8]. The second line drugs used were capreomycin, kanamycin, ethionamide, and ofloxacin. One loopful of MTB colonies was added to 4.5 mL of 20% Tween 80 containing glass beads and placed on a vortex for 1-2 mins until a turbidity equal to number 1 McFarland's standard was obtained. A volume of 0.5 mL of the suspension was used to make serial dilutions of 10^−1^, 10^−2^, 10^−3^, and 10^−4^ for each isolate. The 10^−4^ and 10^−3^ serial dilutions were inoculated onto two plain media quadrants (control) and the 10^−2^ dilution was used to inoculate the quadrants with the drug filled media. Each inoculated Petri dish was sealed in a polythene bag to maintain moisture, incubated at 37°C for a maximum of 4 weeks, and checked for growth weekly. Growth was indicated by the presence of smooth straw colored colonies. Resistance to drugs was interpreted as growth on drug filled media that was significantly more than growth of the 10^−3^ and 10^−4^ serial dilutions of the same isolate on drug-free media.

### 2.5. DNA Extraction

To extract genomic DNA for use in spoligotyping, the frozen regrown MDR-TB isolates were defrosted on a thermomixer at 60°C. 70 *μ*L of 10% SDS (Sigma, USA) and 50 *μ*L of 10 mg/mL proteinase K (Sigma, USA) were added to the thawed sample and incubated for 1 hour at 60°C at 400 rpm on a thermomixer. 100 *μ*L of 5 M NaCl and cetyltrimethylammonium bromide (CTAB) (preheated to 60°C in water bath) was added and mixed by inversion before further incubation for 1 hour at 60°C and 400 rpm. The mix was then incubated at −70°C for 15 minutes before being allowed to thaw and reincubated for 15 minutes at 60°C and 400 rpm. 700 *μ*L of chloroform/isoamyl alcohol (Sigma, USA) (24 : 1) was added and the mixture was centrifuged for 10 mins at 13,000 rpm. The resulting upper aqueous phase was transferred into a clean tube containing 700 *μ*L of cold isopropanol (Sigma, USA) and incubated at 4°C overnight. The next day the solution was centrifuged for 10 minutes at 13,000 rpm and the supernatant was discarded. The tube was washed with 100 *μ*L of 80% ethanol (Sigma, USA) by centrifuging for 10 minutes at 13,000 rpm. The supernatant was discarded and the pellet DNA pellet was air-dried before being resuspended in 50 *μ*L of DNase-free water and kept at −40°C.

### 2.6. Spoligotyping

Spoligotyping of the MDR-TB isolates was performed using the spoligotyping kit from Ocimum BioSolutions (India) as previously described [9]. The spoligotype signatures that were generated were coded into binary format using the following codes: 1 = black dot and 0 = no black dot. The binary codes generated were entered into an Excel spreadsheet and compared with a published online spoligotype database MIRU-VNTRplus [10] where a search by similarity was performed to assign spoligotype based strain lineages and to search for similar types in the database.

## 3. Results

A total of 86 MDR-TB isolates were identified and retrieved from the BRTI/NMRL TB archive. Of these 42 (49%) were isolated from routine specimens (sent in from government referral hospitals) and 44 (51%) were isolated from study specimens (sent in from TB research study sites). Laboratory records of all 86 identified isolates were investigated for geodemographic data. Twenty (23%) isolates failed to grow after subculture onto fresh LJ media, and these were excluded from second line DST and spoligotyping experiments.

### 3.1. MDR-TB Patient Demographics

The study population consisted of 32 (37%) males and 36 (42%) females, with gender data not available for 18 (20.9%) of the patients. The median age and interquartile range (IQR) was 33 (25–40) years, with data on age missing for 23% of the patients. A total of 29 (34%) isolates were obtained from patients residing in Harare Metropolitan province; 11 (13%) were isolated from Manicaland province, 7 (8%) from Mashonaland West province, 8 (9%) from Mashonaland West province, 4 (5%) from Midlands province, 1 (1%) each from Matabeleland North and Bulawayo Metropolitan provinces, and 5 (6%) from patients in Matabeleland South province (see Table 1).

Fourteen (16%) of the isolates were resistant to isoniazid and rifampicin alone, 4 (5%) were resistant to isoniazid, rifampicin, and ethambutol only, and 7 (8%) were resistant to isoniazid, rifampicin, and streptomycin only, with 46 (54%) being resistant to all four first line TB drugs. New TB cases accounted for 3 (4%) of the isolates, with 33 (38%) coming from treatment failure cases, 7 (8%) from TB relapse cases, and 1 (1%) from a treatment defaulter.

A total of 66 (77%) isolates were successfully subcultured onto LJ and subsequently tested for second line drug resistance. No resistance to any of the second line drugs was seen in 48 (73%) of the isolates. Resistance to kanamycin and ethionamide was seen in 3 (5%) of the isolates, whilst 1 (2%) isolate was resistant to ethionamide and ofloxacin. Fourteen isolates (21%) were resistant to ethionamide only.

### 3.2. Spoligotyping

A total of 24 SITs were identified and 18 (69%) matched preexisting shared types in the database. The remaining 6 shared types did not match any isolates in the MIRU-VNTRplus database [10]. The majority (11/18) of the identified shared types were clustered (2 or more isolates with same SIT), and of these 4 predominant (>6 isolates per shared type) clusters were identified. The largest were SIT 1468 (LAM 11-ZWE) with 12 (18%) isolates, SIT 1 (Beijing) with 8 (12%), SIT 60 (LAM 4) with 7 (11%), and SIT 53 (T1) with 6 (9%) isolates (Table 2). These 4 predominant shared types account for half of all the isolates that were genotyped. Three isolates with orphan shared types were identified as belonging to the East African Indian family, 3 from the LAM family, 1 from the Delhi/CAS family, and 1 from the Uganda 1 family.

### 3.3. Measures of Association

Table 3 shows spoligotype based MTB strain lineages stratified by 1st line DST pattern and TB treatment history. There was no significant association between MTB strain lineages and 1st line DST pattern (*P* value 0.57) and between MTB strain lineages and TB treatment history (*P* value 0.88).

## 4. Discussion

The emergence of MDR-TB and XDR-TB is a major global health issue, as high rates of DR-TB have serious consequences for TB control activities, especially in high TB and HIV burden settings, where high mortality and morbidity have been strongly associated with XDR-TB/HIV coinfection. In 2011, global TB data reported an estimated 9.0% of all MDR-TB cases where XDR-TB however very little data is available on DR-TB in Africa due to limited capacity of laboratories in that region to perform DST. Considering that Africa contains 25% of the global TB burden, 80% of which is HIV coinfected, data on DR-TB prevalence in the region is thus essential to guide planning of TB control and management policies.

The TB patients whose MDR-TB isolates were analyzed in this study were predominately young, with a median age of 33 years (IQR: 25–40). Other studies in Africa show a similar age distribution [11, 12] among MDR-TB patients. Studies in Asia also report similar findings with MDR-TB patients having a mean age of 33. Drug resistance data stratified by age is very important in surveillance of drug resistant trends in a setting since a high proportion of drug resistant cases in young age groups could be indicative of recent transmission. In older age groups, high proportions of drug resistance could be an indicator of reactivation of old infections.

In this study, high proportion of “request for examination” forms omitted data on age (23.3%), gender (20.9%), geographic origin (24%), and TB treatment history (48.8%), highlighting poor data collection by nurses and clinicians requesting TB examination for their patients. This presents a challenge to effective monitoring and surveillance of drug resistance patterns and trends, as patient biographical and clinical data is essential in understanding the factors driving TB and possible risk factors for the disease. Effective reporting of such data reduces the need for resource limited countries, such as Zimbabwe, to conduct periodical surveys, which are costly and labour intensive. Routinely collected data is an easy and efficacious way of monitoring TB drug resistance patterns and trends over time, without the need for expensive surveys.

In our current study, the largest group (38%) of MDR-TB isolates was from TB treatment failure cases. By the end of 2012, it was estimated that 20% of global MDR-TB cases were from previously treated TB cases [1]. The proportion of MDR-TB among previously treated cases shown by this study is higher than the global average and this could be explained by a number of factors. The MDR-TB isolates analyzed in this study were archived between January 2007 and December 2011, a period during which Zimbabwe was going through an economic decline. This economic crisis affected health service delivery in the country. Shortage of drugs and trained personnel could have led to the high number of TB patients failing treatment. Zimbabwe is also a high HIV burden nation, with more than 70% of TB cases in the country being HIV coinfected. Studies have shown that, due to the immune compromised state of HIV patients, they are likely to have recurrent TB episodes. HIV patients receiving ART are also at high risk of poor TB treatment adherence due to the high pill burden they take. Some ART drugs have also been documented to have adverse outcomes when administered with anti-TB drugs. These factors combined increase the risk of failing treatment and developing MDR-TB.

A large proportion (53%) of the MDR-TB isolates showed resistance to all four first line drugs (isoniazid, rifampicin, streptomycin, and ethambutol). Poor patient adherence and interrupted drug supplies have been shown to contribute to the emergence of MDR-TB strains. Patients with such organisms pose a challenge for management and treatment to National TB programs. The internationally accepted practice when treating MDR-TB cases is to base the second line regimen on DST results [13]. Having a large proportion of MDR-TB patients resistant to all 1st line drugs places a huge financial burden on the nation as 2nd line drugs are often more expensive, require extended treatment periods, and are often toxic [13–15].

In this study, high proportion of “request for examination” forms omitted data on age (23.3%), gender (20.9%), and TB treatment history (48.8%), highlighting poor data collection by nurses and clinicians requesting TB examination for their patients. This presents a challenge to effective monitoring and surveillance of drug resistance patterns and trends, as patient biographical and clinical data is critical in understanding the factors driving TB and possible risk factors for the disease. Effective reporting of such data reduces the need for resource limited countries, such as Zimbabwe, to conduct periodical surveys, which are costly and labour intensive. Routinely collected data is an easy and efficacious way of monitoring TB drug resistance patterns and trends over time, without the need for expensive surveys.

XDR-TB strains are defined as MTB strains that are resistant to isoniazid and rifampicin (i.e., MDR-TB), plus resistance to a fluoroquinolone and any second line anti-TB injectable aminoglycoside. In this study, the drugs evaluated were ofloxacin, aminoglycosides, kanamycin, and capreomycin. Therefore in the context of our study an XDR-TB strain would have been resistant either to kanamycin and ofloxacin or to capreomycin and ofloxacin. Our study did not identify such resistance patterns, and so there were no XDR-TB strains. A large proportion (73%) of the MDR-TB isolates showed susceptibility to all 2nd line drugs. The remaining 27% of the isolates were shown to be resistant to one or more of the 2nd line drugs but did not meet the definition of XDR-TB. TB strains that are resistant to isoniazid and rifampicin and either a fluoroquinolone or an aminoglycoside, but not both, are termed “pre-XDR-TB” strains.

By late 2011, all of Zimbabwe's neighbors (Botswana, Mozambique, South Africa, and Zambia) had identified at least one XDR-TB strain [1]. Due to the economic situation in Zimbabwe between 2007 and 2009, there was a lot of population movement to and from her neighbors, especially South Africa which is one of the high MDR-TB burden countries and has reported a number of XDR-TB outbreaks [12, 16–18]. Therefore this study hypothesizes that XDR-TB strains could have been imported into the country during this period and thus would be identified among MDR-TB isolates archived during that time. However this study's failure to identify any XDR-TB strains could be due to a number of factors.

There has been much debate on the diagnostic accuracy and reproducibility of some of the methods used to perform 2nd line DST [19–21]. This lack of consensus has led to the absence of an absolute gold standard for 2nd line DST. Second line DST is especially difficult since the critical concentrations of some of the 2nd line drugs are very close to the minimum inhibitory concentrations (MICs); hence the changes in MIC associated with resistance are very small [20, 22, 23]. A lot of research is needed towards standardizing the 2nd line MICs for the various DST methods available. The proportion method on 7H10 Middlebrook has been the gold standard for 2nd line DST in Europe and in America in the past 20 years, but recent WHO guidelines on 2nd line DST recommend the MGIT based DST method [13, 24–26].

This study showed a large proportion (27%) of pre-XDR-TB strains. Globally, the number of pre-XDR-TB strains being identified has increased. A recent study in Nigeria showed a pre-XDR prevalence of 17% [11]. Another study in India reported a pre-XDR-TB prevalence of 42% among MDR-TB cases [27]. The emergence of pre-XDR-TB is a major concern to the TB control program in Zimbabwe, as this highlights possible effects of the use of FQs and aminoglycosides in the treatment of nontubercular infections. However, the majority (14/18) of the pre-XDR-TB strains identified in the study were resistant to ethionamide. There are a number of issues concerning the use of ethionamide in 2nd line DST. The MIC of ethionamide has yet to be standardized [23, 28] and thus the reproducibility of its DST results is questionable. Also the thermoliable nature of ethionamide makes it prone to degradation during media preparation, thus increasing the likelihood of false positive results.

This study also related genetic diversity to the sample of MDR-TB isolates determined by genotyping. A total of 66 MDR-TB isolates were typed using spoligotyping and presented the LAM spoligotype family as the predominant strain type (50%), followed by the T family (13.6%) and finally by the Beijing family (11.9%). The LAM family showed the highest diversity with 9 SITs, LAM 11 ZWE (SIT 1468) being the predominant with 17.9%. The T family had 3 SITs (73, 53, 37), and the Beijing family strains all had SIT 1.

The predominance of the LAM 11 ZWE (SIT 1468) was expected as this SIT has been described as the predominant strain circulating in Zimbabwe by previous studies [29] while the LAM spoligotype family has also been described as the predominant strain circulating in countries neighboring Zimbabwe [7, 29–31]. The presence of the T family was also expected as it is one of the 3 strains reported by the literature to predominate TB infection in Africa [32], along with the Haarlem and LAM families. Studies describing diversity of MTB strain circulating in different parts of Africa also show similar predominance of the LAM spoligotype [33–35], though having different SITs.

Interestingly, the Beijing family had not been seen before in studies describing MTB strains circulating in Zimbabwe. This study however is expected to observe Beijing strains, as the global distribution of this strain is reported to be on the increase [36, 37]. This strain type has also been reported in countries neighboring Zimbabwe, with South Africa reporting a significant proportion of these strains having been isolated from MDR/XDR-TB cases [31, 38].

Much has been postulated in recent publications regarding the association between the Beijing strain and drug resistance [39–42], with observations showing increased bacterial fitness of drug resistant Beijing strains [39, 40]. However, mutations on the MTB genome causing drug resistance on Beijing strains result in reduced fitness cost [41, 43]. This reduced fitness cost explains why the Beijing strain has not taken over as the predominant strain in most drug resistant TB outbreaks and TB endemic populations. Interestingly this study showed that all strains classified as Beijing had the same SIT, which might show the same source of infection, or that the Beijing strain circulating in Zimbabwe is highly conserved resulting in low diversity of the family.

This study showed no significant associations when MTB strain types were stratified by TB treatment history (*P* = 0.88) and first line drug susceptibility profile (*P* = 0.57). These results were expected as no significant association between a particular strain type and drug resistance has been reported. The study was also limited in its power to detect a significant association between strain type and drug resistance due to the small sample size used in this study.

## 5. Conclusion

Despite the small sample size to generalize the study results, this study failed to identify any XDR-TB isolates, but the high proportion (27%) of pre-XDR-TB could be a possible indicator of the future emergence of XDR-TB in Zimbabwe. The SITs described in this study were not different from those reported previously in Zimbabwe. The study also failed to identify SITs reported to have caused XDR-TB and MDR-TB in neighboring countries.

## Figures and Tables

**Table 1:** MDR patient demographics.

Characteristics	Total *n* (%)	Routine specimens *n* (%)	Study specimens *n* (%)
	86	42 (48.8)	44 (51.2)

Gender			
Male	32 (37.2)	14 (33.3)	18 (40.9)
Age			
Median (Q1–Q3)	33 (25–40)	35 (31–45)	27.5 (24–35)
1st line resistance profile			
H + R only	24 (29.7)	4 (10.5)	20 (45.5)
H + R + E only	5 (6.1)	4 (10.5)	1 (2.3)
H + R + S only	8 (9.8)	5 (13.2)	3 (6.8)
H + R + E + S	45 (54.5)	25 (65.8)	20 (45.5)
TB treatment history			
New TB cases	3 (3.5)	—	3 (6.8)
Treatment failure case	33 (38.4)	17 (40.5)	16 (36.4)
TB relapse case	7 (8.1)	4 (9.5)	3 (6.8)
Treatment defaulter	1 (1.2)	—	1 (2.3)

H: isoniazid, R: rifampicin, E: ethambutol, and S: streptomycin.

**Table 2:** Spoligotyping signatures of MDR-TB isolates.

Shared international type (SIT)	Subclade	Octal codes	Total *n* (%)
1	Beijing	000000000003771	8 (11.9)
21	CAS1 KILI	703377400001771	2 (3.0)
25	CAS1 Delhi	703777740003171	1 (1.5)
34	S	776377777760771	3 (4.5)
37	T 3	777737777760771	2 (3.0)
42	LAM 9	777777607760771	3 (4.5)
53	T 1	777777777760771	6 (9.0)
54	MANU 2	777777777763771	1 (1.5)
59	LAM 11-ZWE	777777606060771	2 (3.0)
60	LAM 4	777777607760731	7 (10.5)
73	T 2	777737777760731	1 (1.5)
95	LAM 6	777777607560731	4 (6.0)
398	LAM 9	777777607760631	1 (1.5)
583	MANU 2	777737777763771	1 (1.5)
753	LAM 9	477777607760771	2 (3.0)
813	LAM 11-ZWE	777777606060631	1 (1.5)
1466	LAM 11-ZWE	757777606060771	1 (1.5)
1468	LAM 11-ZWE	077777606060671	12 (17.9)

**Table 3:** MTB strain lineages stratified by 1st line DST and TB treatment history.

	Total *n* (%)	Indo-Oceanic *n* (%)	East Asian *n* (%)	East African Indian *n* (%)	Euro-American *n* (%)	*P* value
1st line DST pattern^#^						
HR resistant	19 (28.4)	1 (5.3)	2 (10.5)	3 (15.8)	13 (68.4)	0.57
HRE resistant	4 (6.0)	1 (25.0)	—	—	3 (75.0)	
HRS resistant	5 (7.5)	—	—	1 (20.0)	4 (80.0)	
HRES resistant	38 (56.7)	1 (2.6)	6 (15.8)	—	30 (78.9)	
TB treatment history*						
New TB cases	3 (4.5)	—	—	—	3 (5.9)	0.88
Treatment failure	27 (40.3)	1 (3.7)	2 (7.4)	3 (11.1)	20 (74.1)	
TB relapse	3 (4.5)	—	—	—	3 (5.9)	

^#^ *n* = 1 missing, *n = 34 missing, H: isoniazid, R: rifampicin, E: ethambutol, and S: streptomycin.
